# Hyperuricemia causes kidney damage by promoting autophagy and NLRP3-mediated inflammation in rats with urate oxidase deficiency

**DOI:** 10.1242/dmm.048041

**Published:** 2021-03-24

**Authors:** Mian Wu, Yiwen Ma, Xiaoting Chen, Nan Liang, Shen Qu, Haibing Chen

**Affiliations:** 1 Department of Endocrinology and Metabolism, Shanghai 10th People's Hospital, Tongji University, Shanghai 200072, China; 2 Department of Endocrinology and Metabolism, The Affiliated Suzhou Hospital of Nanjing Medical University, Suzhou Municipal Hospital, Suzhou 215000, China; 3 Shanghai Diabetes Institute, Shanghai Key Laboratory of Diabetes Mellitus, Shanghai Clinical Center for Diabetes, Department of Endocrinology and Metabolism, Shanghai JiaoTong University Affiliated Sixth People's Hospital, Shanghai 200030, China

**Keywords:** Hyperuricemia, Nephropathy, Autophagy, Inflammation, NLRP3

## Abstract

Epidemiological research has shown that elevated serum urate concentration is a risk factor for the development of kidney disease; however, the mechanisms underlying this process have not yet been elucidated. To examine the role of urate in the kidney, we used Wistar rats to functionally disrupt expression of urate oxidase (UOX) by using the CRISPR/Cas9 system. In comparison to wild-type (WT) rats, serum urate levels spontaneously and persistently increased in *UOX*-KO rats, without showing a significant decrease in survival rate. Architecture and function of the kidneys in *UOX*-KO rats were impaired. Injury to the kidney resulted in increased interstitial fibrosis, macrophage infiltration, increased expression of NLRP3 and IL-1β, and activation of multiple cell-signaling pathways associated with autophagy, such as AMPK, p38 MAPK, ERK and JNK pathways. Inhibition of autophagy with the PI3K inhibitor 3-MA abrogated the development of kidney damage and attenuated renal fibrosis, macrophage infiltration, and expression of NLRP3 and IL-1β in injured kidneys. In conclusion, the *UOX*-KO rat is a great model to study hyperuricemia-related diseases. Hyperuricemia-induced autophagy and NLRP3-dependent inflammation are critically involved in the development of renal damage and, therefore, highlight the inhibition of autophagy and inflammation in search of therapeutic strategies to treat uric acid nephropathy.

## Abstract (precis)

**Summary:** We established a RISPR/Cas9-mediated *Uox* gene-KO Wistar rat model of hyperuricemia, and show that autophagy and NLRP3-related inflammation are involved in uric acid nephropathy.

## INTRODUCTION

Hyperuricemia is a metabolic disease caused by abnormalities in purine metabolism, mainly due to the increased formation or reduced excretion of urate. Approximately two-thirds of the urate produced in humans is excreted by the kidneys. Urate undergoes extensive filtration, reabsorption and secretion in the renal proximal tubule (Jalal, 2016). An increasing number of experimental and epidemiological studies have suggested that uric acid is an independent risk factor for renal disease (Mallat et al., 2016; Obermayr et al., 2008). However, the mechanisms underlying uric acid nephropathy (UAN) remain incompletely understood.

Hyperuricemia may induce renal inflammation through pathways that do or do not involve deposition of crystals, i.e. renal calculi (Braga et al., 2020). It is generally accepted that hyperuricemia induces renal inflammation in a crystal-dependent manner. Monosodium urate (MSU) crystals deposited in the tubular lumen or interstitial space can be recognized and engulfed by macrophages that reside or infiltrate the renal system (Liu et al., 2015; Zhou et al., 2012). Stimulation with MSU crystals produces chemokines, such as CXCL-12, which induce directional proinflammatory cytokines, such as interleukin (IL)-1β (IL1B), IL18 and interferons through the Src/Pyk2/PI3K signaling pathway (Valimaki et al., 2013). Nod-like receptor pyrin domain-containing protein 3 (NLRP3), an important member of the NOD-like receptor (NLR) family, senses danger signals – including pathogen-associated molecular patterns (PAMPs) and damage-associated molecular patterns (DAMPs) – in the cytosol and activates sterile inflammation (Chen and Chen, 2018; Kim et al., 2015). Accordingly, the NLRP3 inflammasome was shown to be upregulated in both UAN patients and in a rat model of UAN (Hu et al., 2019, 2018).

Autophagy is a crucial and rudimentary biological process involved in both physiological and pathological conditions (Liu et al., 2016). Physiologically, it is the degrading process of proteins and organelles mediated by lysosomes, and participates in the regulation of cell metabolism and survival (Liu et al., 2016). Important proteins in the autophagosome membrane mainly include microtubule-associated protein 1 light chain 3 (LC3, officially known as MAP1LC3), beclin-1, and autophagy-related 7 and 12 (Atg7 and Atg12, respectively) (Havasi and Dong, 2016). Many studies have shown that a number of signal transduction pathways are involved in the regulation of autophagy, i.e. PI3K/AKT, MEK–ERK (officially known as MAP2K1/2–MAPK3/1, respectively), and the signaling crosstalk between mTOR cellular and the energy sensor AMP-activated protein kinase (AMPK) that controls of mTOR activity (Wang and Zhang, 2019). Recent studies have confirmed that autophagy also plays a functional role in hyperuricemia-induced inflammation (Maejima et al., 2013; Saitoh et al., 2008). Activation of autophagy may limit inflammasome activity induced by hyperuricemia by targeting ubiquitylated inflammasomes for degradation (Shi et al., 2012) and decreasing the production of reactive oxygen species (Isaka et al., 2016) and downstream inflammatory responses (Choe et al., 2014). This may be why gouty arthritis is self-limiting. In UAN, however, there have been contradictory reports regarding autophagy. One study has shown that persistent inhibition of autophagy promotes renal damage in rats with UAN, when fed a mixture of adenine (0.1 g/kg) and potassium oxonate (1.5 g/kg) daily for 3 weeks (Bao et al., 2018), whereas another study showed that upregulation of autophagy by Weicao exerts anti-inflammatory and renal protective effects in UAN rats (Hu et al., 2018). These discrepancies may have been due to the differences is modeling schemes between the studies. Therefore, a suitable model to confirming the role of autophagy and related inflammation in UAN is necessary.

In most mammals, serum urate concentrations are low [1–3 mg/dl (60–180 μM)], because the enzyme urate oxidase (UOX, also known as uricase), degrades uric acid to 5-hydroxyisourate and, eventually, to allantoin. Owing to inactivating mutations that occurred during hominoid evolution, humans and apes lack uricase, resulting in higher circulating urate concentrations in these species. At present, drug induction through purine synthesis promoters and/or uricase inhibitors are the primary strategy to establish hyperuricemia models. However, serum urate levels are often unstable and can fluctuate widely in animal models of drug-induced hyperuricemia. Alternatively, a hyperuricemia model established by using urate oxidase gene knockout (KO) leads to significant hyperuricemia, but few of these animals survive to maturity (Zhu et al., 2017). Encouragingly, Lu et al. generated a *UOX*-KO mouse with a pure C57BL/6J genetic background by using transcription activator-like effector nuclease (TALEN) technology (Lu et al., 2018). The laboratory rat, *Rattus norvegicus*, has been used as an animal model for human diseases for more than 150 years. Its size, together with its cognitive and physiological characteristics make the rat a useful model for cardiovascular disease, neurological disorders and metabolic disorders. Rat models are superior to mouse models for testing pharmacodynamics, pharmacokinetics and toxicity, partly because many of their detoxifying enzymes are very similar to those in humans (Alcantar and Rairdan, 2019). Therefore, there is a need for rat models that are not only easy to establish but also more representative of human pathologies. The clustered regularly interspaced short palindromic repeats (CRISPR)/CRISPR-associated protein (Cas) technology has provided a much simpler and more economical method for gene-targeted modification. This engineered nuclease generates a DNA double-strand break (DSB) at the targeted genome locus. The break activates a repair mechanism through error-prone non-homologous end joining (NHEJ) or homology-directed repair (HDR). In the absence of a template, NHEJ is activated, resulting in insertions and/or deletions (indels) that disrupt the target loci. In the presence of a donor template that is homologous to the targeted locus, the HDR pathway operates, thereby allowing for precise mutations to be made (Ma et al., 2014). CRISPR/Cas9 is a simple and efficient method to generate precise genetic modifications in rats, which will promote the accumulation of genetic resources in rats and enable more precise studies of gene function (Alcantar and Rairdan, 2019).

In this study, a novel hyperuricemia model was established in Wistar rats through *Uox* gene KO after using the CRISPR/Cas9 system. By using this model, we demonstrated that hyperuricemia induces renal damage together with tubular injury and kidney fibrosis through activation of autophagy and NLRP3 inflammasome-mediated inflammation.

## RESULTS

### Generation of UOX-KO rats with hyperuricemia

**Fig. 1.**
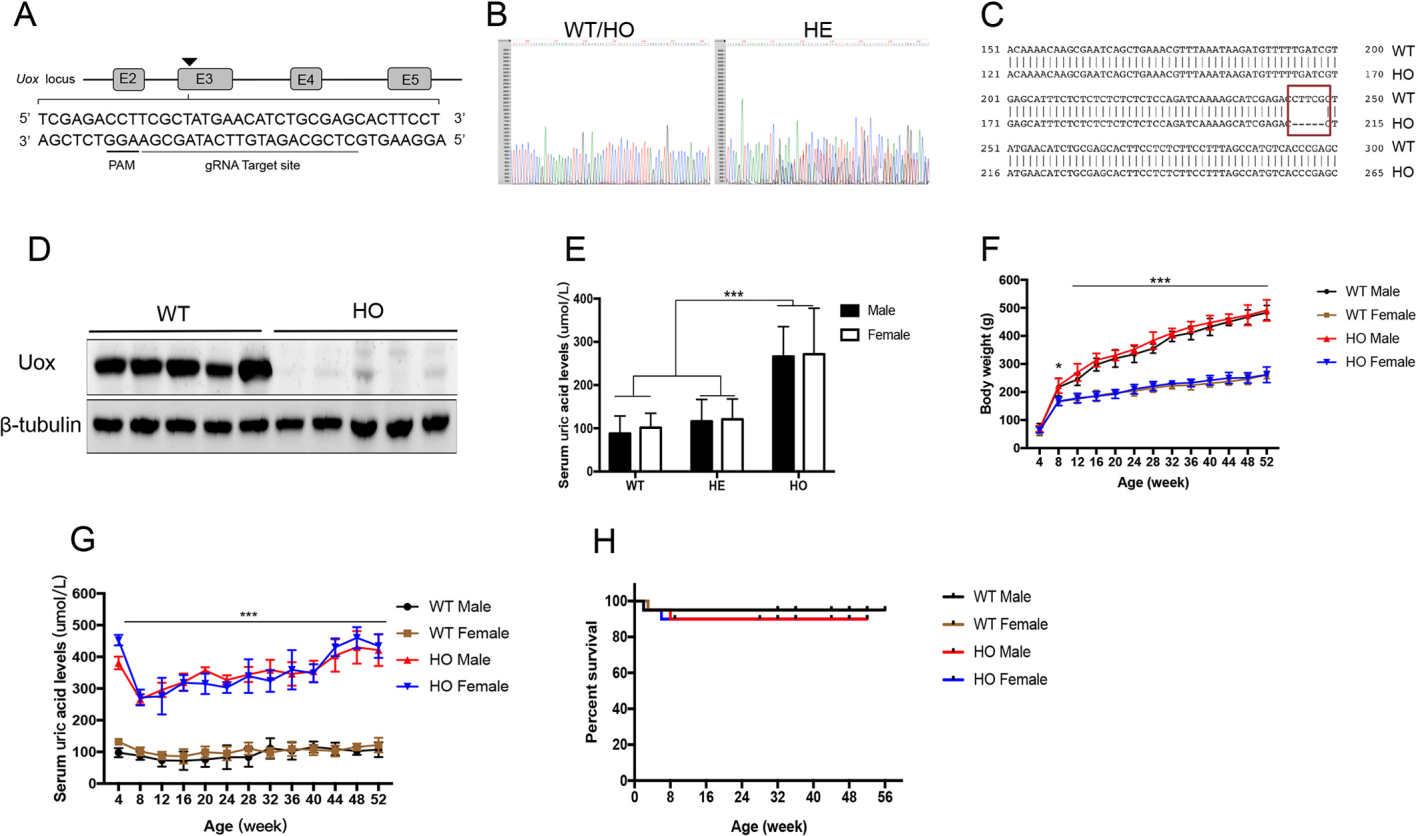
**Generation of *UOX*-knockout (KO) rats with hyperuricemia.** (A) Schematic representation of *UOX* KO using CRISPR/Cas9. (Top) *Uox* locus, boxes indicate exons and connecting lines indicate introns. (Bottom) Portion of the *Uox* sequence, showing the gene editing site (gray underlined), and the protospacer adjacent motif (PAM) region (black underlined). (B) Sanger sequencing of the PCR-amplified genomic *Uox* locus revealed DNA disruption at the gRNA-target site downstream of the Cas9 cleavage site in rats heterozygous (HE) for *Uox* KO, but not in wild-type (WT) rats or in rats homozygous (HO) for *Uox* KO. (C) Schematic targeting and deep sequencing reads of the *Uox*-locus with deletions (red box) in HO rats. (D) Hepatic expression of UOX protein determined by western blotting (8-week-old males, *n*=5). (E) Serum urate levels of WT, HE and HO rats, of both sexes (8 weeks of age, *n*=5 per group). (F) Body weight of male and female WT or HO rats (aged between 4 and 52 weeks), measured every 4 weeks (*n*=5 per group). (G) Serum urate levels of male and female WT and HO rats (aged between 4 and 52 weeks), measured every 4 weeks (*n*=5 per group). (H) Kaplan–Meier plot, showing the survival rates of HO (male, *n*=50; female, *n*=50) vs WT rats (male, *n*=50; female, *n*=50) from birth to 56 weeks of age. Values are presented as the mean±s.d. *P<0.05, ***P<0.001.

The KO scheme is shown in Fig. 1A. The rat *Uox* gene consists of nine exons. We designed a 20-nucleotide-long targeting guide RNA (gRNA) construct (gRNA^TRGT^) complementary to a region in exon 3, immediately upstream of the protospacer adjacent motif (PAM) unique to the *Uox* locus, to discriminate between alleles during Cas9 cleavage. Genomic DNA was extracted from pups for Sanger sequencing. Multiple peaks from samples of rats heterozygous (HE) for *Uox* KO (HE rats) indicated the presence of genomic sequences originating downstream of the disparate PAM of *Uox*, whereas samples from rats homozygous (HO) for *Uox* KO (HO rats) and from WT rats produced single peaks (Fig. 1B). Further alignment with WT rat sequences indicated that HO rats carried a 5-bp deletion in the *Uox* gene (Fig. 1C). Expression of uricase in the liver was significantly lower in HO than in WT rats (Fig. 1D).

HO rats had significantly higher serum urate levels compared with those in HE and WT rats at 8 weeks after birth. There were no differences in serum urate levels between male and female rats (Fig. 1E) of that age. Observation was then extended to rats between 4 and 52 weeks of age, and we found no differences in body weight between WT and HO rats of the same sex (Fig. 1F). However, consistently, serum urate levels were significantly higher in HO rats than in WT rats (Fig. 1G). Between 12 and 52 weeks of age, serum urate levels in WT rats stabilized to ∼100 μmol/l, whereas serum urate levels in HO rats gradually increased from 280 to 400 μmol/l. To determine the survival rate of HO rats, we conducted Kaplan–Meier analysis over a 52-week observation period. The results showed that >95% of HO rats survived for >1 year (Fig. 1H). Genotyping at 1–2 weeks after birth revealed that the distribution of WT, HE and HO rats was consistent with Mendelian inheritance, i.e. 29% (72 of 252), 48% (120 of 252), and 24% (60 of 252), respectively. Results of mating between homozygous *Uox*-KO rats showed that the number of births was normal, i.e. ∼8–14 pups per dam, suggesting that loss of the *Uox* gene did not lead to embryonic or neonatal death.

Taken together, these results indicate that the CRISPR/Cas9 system successfully constructed *Uox*-KO rats that exhibited persistent spontaneous hyperuricemia. The transgenic *Uox*-KO rat was named Wistar-Uox^em2Cd5^/IDM. Here, HO rats are referred to as *UOX*-KO rats.

### Renal function was impaired in *UOX*-KO rats

**Fig. 2.**
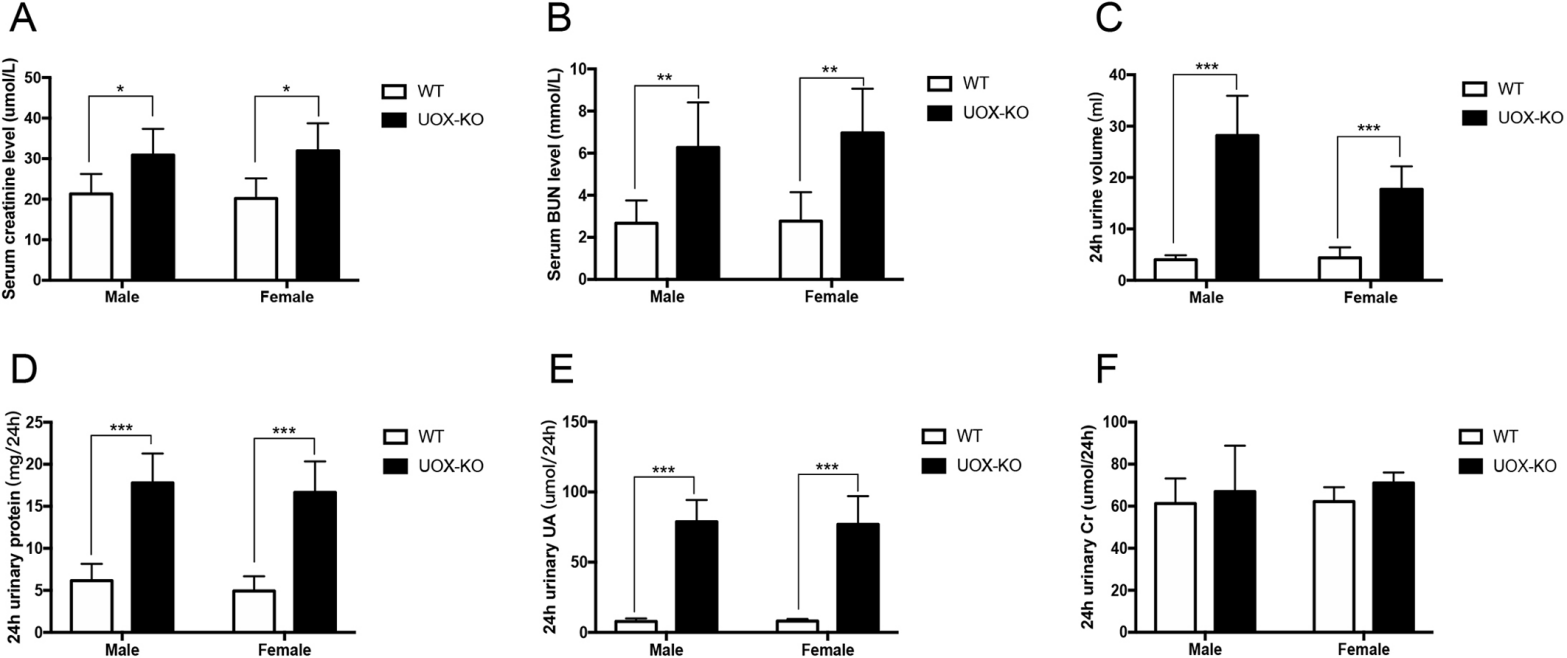
**Renal function was impaired in *UOX*-KO rats.** (A,B) Serum creatinine (A) and urea nitrogen (B) in *UOX*-KO vs WT rats of both sexes. (C–F) Levels of 24-h urine volume (C), urine protein (D), urine uric acid (E) and urine creatinine (F) in *UOX*-KO and WT rats of both sexes at 8 weeks of age (*n*=5 per group). Values are presented as the mean±s.d. *P<0.05, **P<0.01, ***P<0.001.

**Fig. 3.**
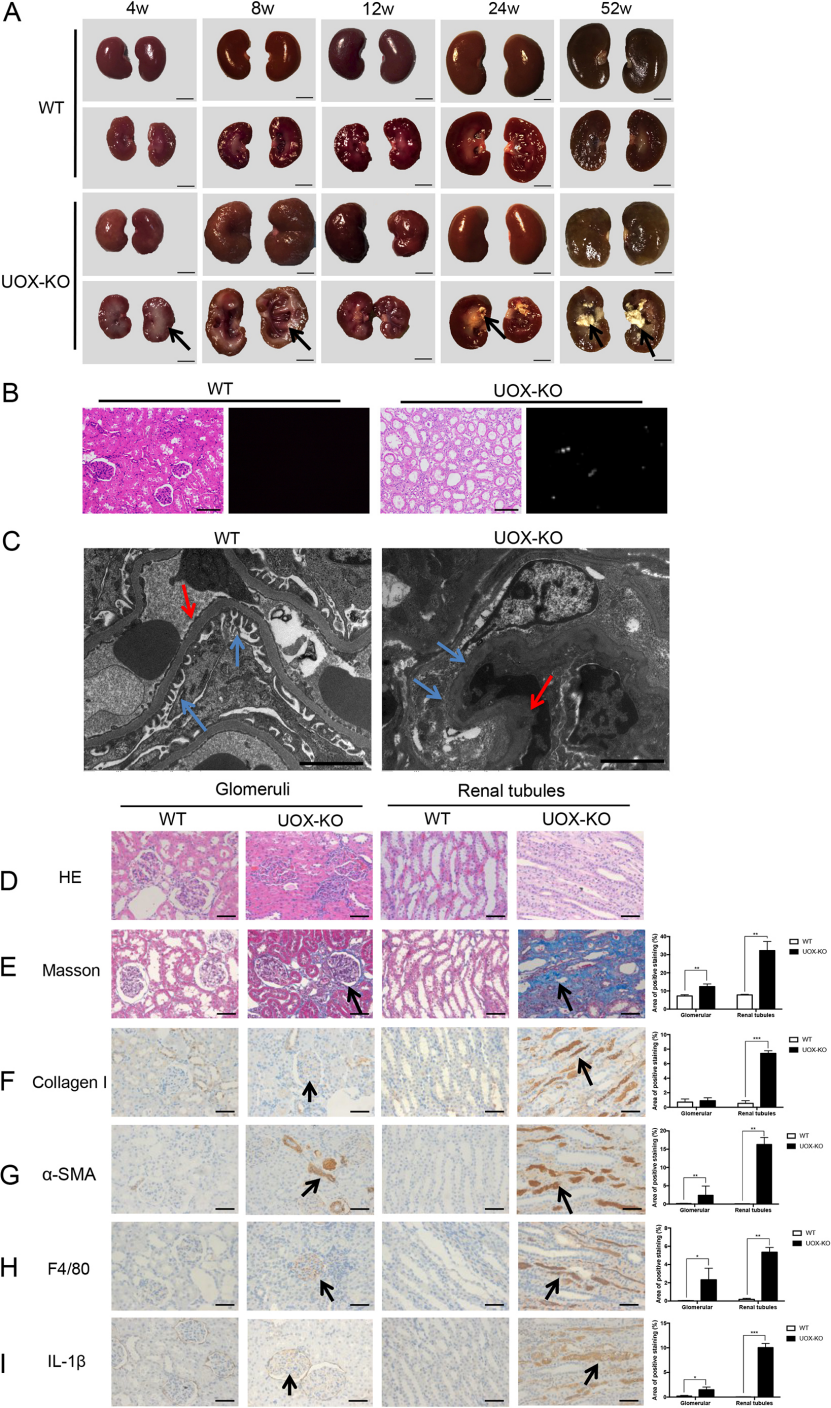
**Renal histopathology was impaired in *UOX*-KO rats.** (A) Images show complete kidneys and longitudinal sections of kidneys from male *UOX*-KO and WT rats as indicated, in weeks (w). Arrows either indicate lesions (4w and 8w) or urate crystals (24w and 52w) in kidneys of UOX-KO rats. (B) H&E staining (right) and polarized light images (left) of kidneys from 16-week-old WT and UOX-KO rats. Urate crystals, detected under polarized light in the kidney of the UOX-KO rat. (C) Transmission electron microscopy of kidney samples from male WT and *UOX*-KO rats at 8 weeks of age. Blue arrows indicate podocytes, red arrows indicate the basement membrane. (D–I, left) Images show kidney tissue collected from 12-week-old WT or UOX-KO rats. Columns one and two show glomeruli, columns three and four show renal tubules. Tissue was analyzed using H&E staining (D) or Masson's trichrome staining (E), or immunostained for the renal expression of collagen I (F), alpha-smooth muscle actin (α-SMA) (G), F4/80 (H) and interleukin (IL)-1β (I) by using corresponding antibodies (*n*=5 per group). Arrows in E–I indicate positive staining. Scale bars: 0.5 cm (A), 200 μm (B), 2 μm (C), 50 μm (D–I). (Right) Bar graphs showing quantification of each analysis. Values are presented as the mean±s.d. *P<0.05, **P<0.01, ***P<0.001.

Renal function was determined at 8 weeks of age in WT and *UOX*-KO rats of both sexes. Compared with WT controls, serum creatinine and blood urea nitrogen (BUN) levels were significantly elevated in both male and female *UOX*-KO rats (Fig. 2A,B). Compared with WT controls of the same sex, the 24-h urine volume of male *UOX*-KO rats increased 7.02-fold (4.01±0.87 ml vs 28.17±7.7 ml), whereas that of female *UOX*-KO rats increased 4.05-fold (4.38±2.04 ml vs 17.72±4.46 ml) (Fig. 3C). Compared with WT controls, the 24-h total urinary protein level also increased significantly in both male and female *UOX*-KO rats (6.15±1.99 mg/24 h vs 17.78±3.51 mg/24 h and 4.92±1.76 mg/24 h vs 16.65±3.51 mg/24 h, respectively) (Fig. 2D). Compared with WT rats, urinary uric-acid excretion increased significantly in *UOX*-KO rats (Fig. 2E), whereas total urine creatinine levels did not differ between groups (Fig. 2F). These data indicate that uric acid can cause severe renal insufficiency and, especially, renal concentration dysfunction.

### Renal histopathology was impaired in *UOX*-KO rats

Kidney morphology was examined at 4–52 weeks of age. Compared with male WT rats, male *UOX*-KO rats exhibited significant impairment of kidney gross morphology, characterized by a less-smooth surface and structural damage (Fig. 3A). Renal swelling and polycystic changes in *UOX*-KO rats occurred at ∼8 weeks of age, followed by crystal deposition in the renal medulla and visible nephrolithiasis from 12–52 weeks of age. Renal histopathology was examined at 16 weeks of age. Urate crystals were detected in the kidney interstices in *UOX*-KO rats under polarized light (Fig. 3B). Transmission electron microscopy revealed renal structural destruction, extensive fusion of podocytes, and thickening of the basement membrane in the kidneys of *UOX*-KO rats at 8 weeks of age (Fig. 3C). Hematoxylin and eosin (H&E) staining of kidney tissues revealed the presence of atrophic glomeruli and tubular ectasia in *UOX*-KO rats (Fig. 3D). Fibrosis is a pathological feature of UAN. Specifically, Masson's trichrome staining as well as staining for collagen I and alpha-smooth muscle actin (α-SMA) revealed that glomerular and renal tubule fibrosis – especially tubulointerstitial fibrosis – were prominent in *UOX*-KO rats compared to WT rats (Fig. 3E–G). Chronic inflammation was also observed in *UOX*-KO rats, manifested as substantial infiltration of macrophages (F4/80) and increased expression of IL-1β, especially in the renal interstitium (Fig. 3H and I, respectively). Taken together, these data indicate that hyperuricemia contributes to histopathological damage, mainly in the renal interstitium.

### Autophagy in the kidney is increased through hyperuricemia

**Fig. 4.**
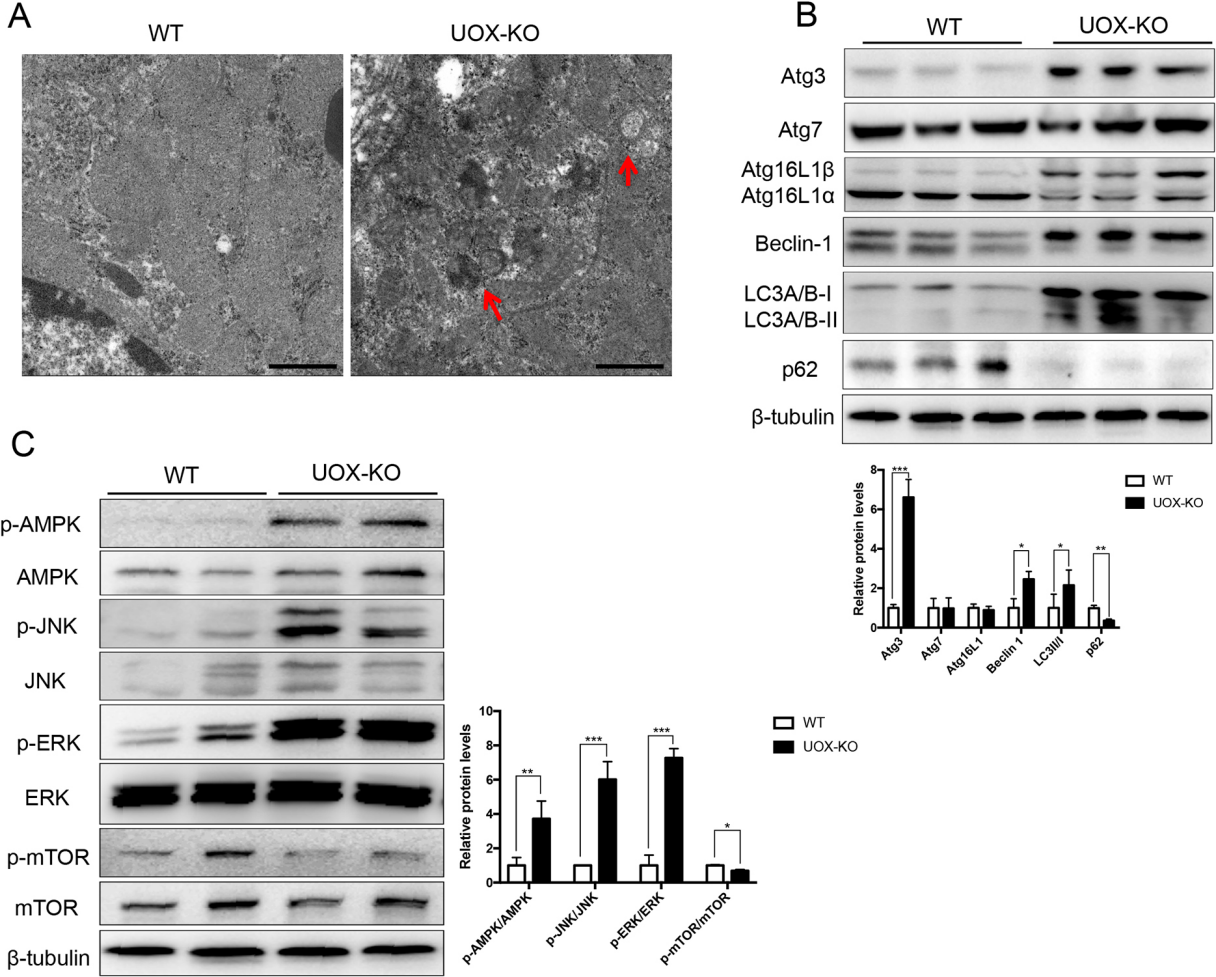
**Hyperuricemia increased autophagy in the kidney.** (A) Samples from 12-week-old male WT and *UOX*-KO rats were examined using scanning electron microscopy. Autophagosomes are indicated by red arrows. (B) Renal expression of autophagy-related proteins determined by western blotting. (C) Renal expression levels of phosphorylated (p-) and total AMPK, JNK, ERK and mTOR proteins determined by western blotting. Scale bars: 1 μm. Data are shown as the mean±s.d. and representative of at least three independent experiments. *P<0.05, **P<0.01, ***P<0.001.

A number of studies have revealed the role of autophagy in kidney diseases. To validate the role of autophagy in UAN, we further examined the appearance of autophagosomes and related autophagic vacuoles by using electron microscopy. Whereas we rarely observed autophagic vacuoles in the kidneys of WT rats, we noticed numerous autophagic vacuoles in the proximal tubular cells of *UOX*-KO rats (Fig. 4A). Subunit light chain 3 (LC3) of microtubule-associated protein 1 light chain 3 (MAP1LC3) isoforms had originally been identified as an autophagy marker. The three human MAP1LC3 isoforms (MAP1LC3A, MAP1LC3B and MAP1LC3C) undergo post-translational modifications during autophagy, i.e. cleavage of their LC3 at the C-terminus immediately after synthesis yields a soluble cytosolic LC3-I form that, during autophagy, is lipidated to LC3-II by a ubiquitin-like system involving Atg3 and Atg7. This allows LC3-II to become associated with autophagic vesicles. The presence of LC3 in autophagosomes and its conversion to the less-migrating form LC3-II is, therefore, an indication of autophagy (Maejima et al., 2013). In this study, autophagy levels were also assessed by determining the LC3II:LC3I ratio, and the levels of beclin-1, p62 and Atg expression. In *UOX*-KO rats with hyperuricemic damage, the expression of beclin-1 and Atg3 and the LC3II:LC3I ratio increased, whereas expression of SQSTM1 (hereafter referred to as p62) in the kidney decreased (Fig. 4B). Autophagy can be regulated by different signaling pathways, such as the AMPK and MAPK/ERK pathways, which inhibit mTOR and then activate autophagy. Therefore, we examined these pathways and found that, in kidneys of *UOX*-KO rats, protein levels of phosphorylated JNK, ERK1/2 and AMPK increased, whereas the levels of phosphorylated mTOR decreased when compared to levels in WT rats (Fig. 4C). These observations indicate that hyperuricemia can induce the formation of autophagic vacuoles, which are related to the regulation of AMPK- and MAPK-related pathways via uric acid.

### Inhibition of autophagy prevented renal dysfunction and alleviated renal histopathological changes in *UOX*-KO rats

**Fig. 5.**
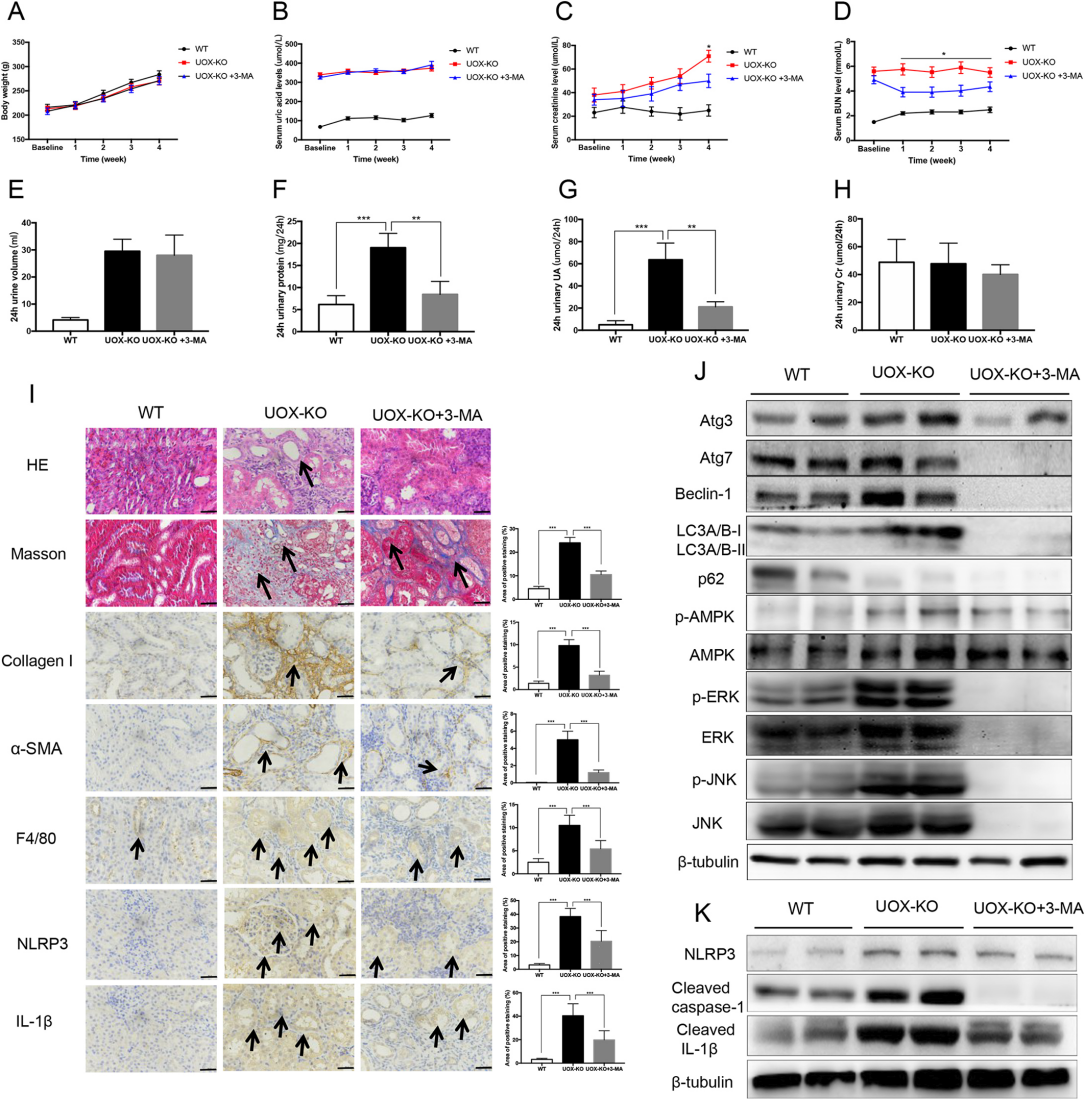
**Inhibition of autophagy prevented renal dysfunction and alleviated renal histopathological changes in *UOX*-KO rats.** Rats were sacrificed at 4 weeks after 3-MA administration. (A–G) Body weight (A), fasting serum uric acid (B), creatinine (C) and blood urea nitrogen (BUN) (D) levels were measured weekly. The 24-h urine volume (E), urine protein (F), urine uric acid (G) and urine creatinine (H) levels were measured at the end of each experiment. (I) Renal histopathology of three groups was carried out using H&E and Masson's trichrome staining. (Left) Images show renal expression of collagen I, α-SMA, F4/80, NLRP3 and IL-1β, determined by immunostaining with respective antibodies. Arrows indicate renal tubule dilation for H&E staining, positive staining for Masson's staining, or immunostaining for expression of collagen I, α-SMA, F4/80, NLRP3, and IL-1β. (Right) Bar graphs showing quantification of each analysis. (J) Renal expressions of autophagy-related proteins and upstream pathways, including those of AMPK, ERK and JNK were determined by western blotting. (K) Renal expression of NLRP3 inflammasome-related proteins. Data in J and K are representative of four independent experiments (*n*=5 per group; A–I). Scale bars: 50 μm. Values are presented as the mean±s.d. *P<0.05, **P<0.01, ***P<0.001.

To further assess the roles of autophagy in UAN, we investigated effects of the PI3K inhibitor 3-methyladenine (3-MA) on renal function and fibrosis in *UOX*-KO rats. There were no significant changes in body weight or serum urate levels in *UOX*-KO rats after 4 weeks of daily feeding with 3-MA (Fig. 5A,B). However, serum creatinine and BUN levels decreased significantly in rats fed 3-MA (Fig. 5C,D). The 24-h urine volume in the 3-MA group did not differ from that of the control *UOX*-KO group (Fig. 5E), whereas the 24-h urinary protein and uric acid levels decreased significantly (Fig. 5F,G). In addition, 24-h urine levels were not markedly altered by 3-MA.

Histopathological changes in the kidney were examined using immunohistochemistry (Fig. 5I). Semi-quantitative analysis of Masson's trichrome-positive areas revealed that there was an ∼5.3-fold increase in the deposition of extracellular matrix (ECM) components in hyperuricemic injured kidneys compared with control kidneys, whereas treatment with 3-MA reduced the deposition of ECM components by 56%. These results were also confirmed when staining for collagen I and α-SMA. Kidney fibrosis is aggravated by chronic inflammation, which is characterized by macrophage infiltration. As shown in Fig. 5I, the number of F4/80-postive macrophages in the injured kidney increased in *UOX*-KO rats compared to WT rats, and 3-MA treatment markedly inhibited the infiltration of these cells. Levels of NLRP3 and IL-1β expression were also significantly reduced by 3-MA treatment. Hyperuricemic damage activated autophagy, characterized by increased LC3II:LC3I ratio and decreased expression of p62; moreover, AMPK, ERK and JNK signaling pathways were also activated. As shown in Fig. 5J, 3-MA inhibited the autophagy process, and Atg3, Atg7, beclin-1 and LC3 were significantly downregulated in 3-MA-treated kidneys. In addition, activation of AMPK, ERK and JNK was also suppressed by 3-MA. Furthermore, 3-MA inhibited the expression of NLRP3, cleaved caspase-1 and cleaved IL-1β (Fig. 5K), suggesting that the inhibition of autophagy improves renal function by reducing NLRP3-mediated inflammation in UAN.

## DISCUSSION

Hyperuricemia is critically associated with chronic kidney injury and the prevalence of UAN has increased worldwide. Here, we described a CRISPR/Cas9-mediated *Uox* gene-KO Wistar rat model of UAN. KO of the *Uox* gene in rats resulted in spontaneously sustained high serum uric acid levels and severe nephropathy, characterized by increased BUN and creatinine levels, increased levels of urinary volume and proteins, renal fibrosis and inflammatory cell infiltration in the kidney. Moreover, we found that autophagy is required for renal tubular injury, and the activation of multiple signaling pathways associated with renal fibrogenesis and inflammation. By using the autophagy inhibitor, 3-MA, we demonstrated that inhibition of autophagy can preserve kidney function, reduce NLRP3-induced inflammatory responses and suppress renal interstitial fibrosis in UAN.

When compared with WT rats, serum uric acid levels were higher in *UOX*-KO rats from as early as 2 weeks after birth, and this increase lasted for >1 year. Previously, Lu et al. had reported that *UOX*-KO C57BL/6J mice have elevated serum urate levels (420–520 μmol/l) that are 2–3-fold higher than those in WT mice (Lu et al., 2018). Other studies that had also used *UOX*-KO mice, serum urate levels varied between 200 and 600 μmol/l (Hosoyamada et al., 2016; Inazawa et al., 2016). Compared with these *UOX*-KO transgenic mouse models, we – using our *UOX*-KO rat model – only observed a mild increase in the levels of serum urate. This may be because the average serum urate level of WT Wistar rats is below that that of WT C57BL/6J mice, i.e. 80 μmol/l vs 200 μmol/l. Furthermore, our *UOX*-KO rats exhibited a high survival rate, with a 1-year survival of >95%, which was significantly higher than that of *UOX*-KO mouse models. These observations indicate that our *UOX*-KO Wistar rat model – producing spontaneous 2–3-fold increases in serum urate levels that were maintained over a long period – can be a useful model for the long-term study of hyperuricemia and its complications.

The most prominent phenotype in the *UOX*-KO rats was kidney damage and severe UAN. Regarding our *UOX*-KO rat model, we were able to establish the following: First, serum biochemical indicators of renal function showed that serum creatinine and BUN levels were elevated. Second, urine tests indicated that urine volume and protein levels had increased. Third, morphological examination revealed polycystic changes in the kidney at 8 weeks of age, and granular and atropic changes at 52 weeks of age in combination with the formation of renal crystals. Fourth, light microscopy revealed tubular dilatation, renal cortex atrophy and glomerular hypertrophy, some extent of glomerulosclerosis, significant tubular fibrosis, and the deposition of urate crystals. Fifth, macrophage infiltration, increased expression of inflammatory factors and apoptosis were seen within the kidney, especially in the renal tubules. Physiological characteristics of uric acid excretion may be responsible for hyperuricemia leading to kidney injury, especially tubular damage. In our *UOX*-KO model, kidney damage occurred at a very early stage; thus, hyperuricemia was likely to be a direct cause of kidney damage.

However, the mechanisms underlying UAN are still unclear. Autophagic responses to kidney damage can be beneficial or harmful, depending on the pathological setting (Kimura et al., 2017). With regard to obstructed kidneys, Livingston et al. (2016) have shown that the persistent activation of autophagy in kidney proximal tubules potentiates renal interstitial fibrosis by promoting overexpression of transforming growth factor beta, tubular cell death as well as interstitial inflammation in a mouse model. However, in a rat model of unilateral ureteral obstruction, Yang et al. (2020) have suggested that autophagy limits fibrosis by abolishing apoptosis. In this study, we demonstrated that chronic insult with uric acid led to the induction of autophagy – represented by increased LC3II:LC3I ratio and beclin-1 expression, and decreased expression of p62 in the kidney. Furthermore, AMPK, ERK and JNK were activated and mTOR was inhibited in the kidneys of *UOX*-KO rats. Bao and colleagues reported that the 3-MA-mediated inhibition of autophagy attenuated hyperuricemic nephropathy in an adenine and potassium oxonate-induced hyperuricemia rat model (Bao et al., 2018). We, consistent with previous research (Bao et al., 2018; Livingston et al., 2016), found that 3-MA improved renal function, decreased urine microalbumin, attenuated pathological changes, inhibited activation of renal interstitial fibroblasts and decreased the accumulation of ECM proteins in the hyperuricemic kidney. These observations imply that autophagy plays an important role in promoting hyperuricemic nephropathy.

MSU acts as a ‘danger signal’, designated as a DAMP, which warns the innate immune system of cellular insult, activates the NLRP3 inflammasome, and produces a proinflammatory response to repair damaged tissues (Martinon et al., 2006). In this study, we demonstrated that hyperuricemia can cause intrarenal crystal formation in the kidney, macrophage infiltration, and upregulation of NLRP3 and IL-1β expression. Autophagy inhibition may protect against hyperuricemic nephropathy by inhibiting proinflammatory responses. Uric acid stimulates the activation of the NLRP3 inflammasome and PI3K/AKT signaling pathway in kidney epithelial cells (Lu et al., 2019) and the intestine (Chen et al., 2018). Tavares and colleagues have reported that, in the absence of PI3Kγ activity, production of cleaved caspase-1 and IL-1β in synovial tissue decreased after the injection of MSU crystals (Tavares et al., 2019). We found here that inhibition of autophagy by the PI3K inhibitor 3-MA downregulated AMPK, ERK and JNK signaling pathways, resulting in a decrease in NLRP3 activity and the subsequent suppression of IL-1β release. These results suggest that autophagy is crucial for UAN, and necessary to regulate NLRP3 activation and IL-1β production. Drugs that regulate autophagy may contribute to the alleviation of renal injury and improvement in renal function.

In summary, we generated a CRISPR/Cas9-based *Uox*-KO Wistar rat model that exhibited persistent and stable hyperuricemia, suggesting it may be an effective and reliable tool to study the mechanisms underlying hyperuricemia-related diseases and their potential treatments. Moreover, *UOX*-KO rats exhibited significant kidney damage, with autophagy activated in the kidney, whereas inhibition of autophagy in response to treatment with 3-MA alleviated UAN by reducing inflammation. Besides the traditional therapy to treat UAN, the regulation of autophagy or the management of urate-induced NLRP3 inflammasomes might be novel effective therapeutic strategies.

## MATERIALS AND METHODS

### Generation of UOX-KO rat

The specific strategy to edit rat *Uox* by using the CRISPR-Cas9 system is described in Fig. 1A. A guide RNA (gRNA) was designed to target exons 3 in the *Uox* gene, based on software tools that predict unique target sites throughout the rat genome (http://crispr.mit.edu/). The gRNA sequence used was 5′-CTCGCAGATGTTCATAGCGA-3′, the PAM sequence was AGG. The gRNA was cloned upstream of the scaffold gRNA sequence into px330 vectors (pX330-U6-Chimeric_BB-CBh-hSpCas; BioVector Science Lab, China) using BbsI restriction enzyme sites. A mixture of transcribed Cas9 and gRNA was microinjected into Wistar rat zygotes. After microinjection, injected zygotes were transferred into pseudopregnant Wistar rats. All rats used in this study were bred in an AAALAC-accredited facility with *ad libitum* access to food and water. All animal experiments were approved by the Animal Research Ethics Committee of the Shanghai Jiaotong University-affiliated Sixth People's Hospital, Shanghai, China.

### Genomic DNA preparation and genotyping

Genomic DNA was extracted from the tails of 7-day-old rats. In brief, each rat tail was dissolved overnight in 400 μl SNET (100 mM Tris-Cl, 25 mM EDTA, 100 mM NaCl, 0.5% SDS, 0.1 mg/ml proteinase K) at 55°C. Genomic DNA was extracted in the lysis mixture by adding 2.5 volumes of ethanol, and dissolved in 200 μl double-distilled H_2_O at 55°C for at least 4 h. PCR primers used for genotyping the modified rats were as follows: UOX-forward primer: 5′-CCCAGGCTAAACTCTCAGGCT-3′; reverse primer: 5′-TGTCAGGGAAACAGTCATTTCACA-3′. PCR reaction (initialization: 93°C for 3 min; denaturation: 40 cycles at 93°C for 30 s, annealing: at 57°C for 30 s, and 65°C for 2 min; and a final extension: at 65°C for 10 min) was performed using Multiplex PCR Mix (novoprotein, China) in an Applied Biosystems Veriti™ 96-well Thermal Cycler (Thermo Fisher Scientific, Waltham, MA, USA). PCR products were used for sequencing analysis and the sequencing primer was UOX-forward primer.

### Quantification of serum uric acid levels and renal function

WT and UOX-KO male and female rats (*n*=5 per group) were used to analyze serum uric acid levels and kidney function. Every 4 weeks, rats were fasted overnight, starting at age 4 weeks and up to age 52 weeks. The next morning, blood was collected from the tail vein, followed by incubation at room temperature for 1 h. Sera were collected and stored at −80°C for biochemical indicators analysis. A Urine sample for each mouse was collected over 24 h in a metabolic cage. Samples were centrifuged at 2000 ***g*** for 10 min to remove particulate contaminants. The supernatant was used to determine uric acid, creatinine, and protein levels.

### Analysis of biochemical indicators

Biochemical indicators, including serum UA, creatinine and BUN were determined by using respective analysis kits (Nanjing Jiancheng Bioengineering Institute, China). 24-h urine was collected for urine creatinine and uric acid detection, by using an automatic biochemical analyzer (Toshiba, Tokyo, Japan). Urine protein levels were measured using a BN2 automatic protein analyzer (Siemens, Germany).

### Drug treatment

Five male WT and ten male UOX-KO rats were used to test the effect of autophagy on uric acid nephropathy. Ten male UOX-KO rats were randomly divided into control or 3-methyladenine (3-MA; 15 mg/kg/day; MCE, China) groups. Both WT and UOX-KO control groups were given normal saline. Intragastric drug (3-MA) and normal saline administration was continuously every 24 h for 4 weeks. Fasting sera were collected every week for analysis of biochemical indicators. 24 h before the final drug administration, the urine sample for each rat was collected. At the end of treatments, kidney tissues were collected for histology or stored at −80°C for western blot analysis.

### Western blotting

Livers or kidneys were lysed using RIPA buffer (Beyotime Biotechnology, China) with 100 μM PMSF and a phosphatase inhibitor cocktail (Roche). Homogenates were centrifuged at 12,000 ***g*** for 15 min, and 40 μg of supernatants protein were separated using standard SDS-PAGE and transferred to PVDF membranes. Blocking was performed using 5% skimmed dry milk for 1 h at room temperature. In general, primary antibodies were obtained from CST (Cell Signaling Technology) and used at 1:1000 in TBST containing 5% BSA. Those antibodies were against Atg3 (#3415), Atg7 (#8558), Atg16L1 (#8089), beclin-1 (#3495), LC3A/B (MAP1LC3A/MAP1LC3B #12741), p62 (SQSTM1, #39749), phosphorylated AMPK (PRKAA #2535), AMPK (#2532), phosphorylated JNK (#4668), JNK1/2/3 (MAPK8/MAPK9/MAPK10 #9252), phosphorylated ERK1/2 (MAPK3/MAPK1#4370), ERK1/2 (#4695), phosphorylated mTOR (#2974), mTOR (#2983) and β-tubulin (#2128). Obtained from other suppliers were antibody against uricase (1:500, sc-166214, Santa Cruz Biotechnology), caspase-1 p10 (1:500, sc-514, Santa Cruz Biotechnology), NLRP3 (1:1000, NBP2-12446, Novus) and IL-1β (1:2000, AF-401-NA, R&D systems). After extensive washes (at least five times) with TBST buffer, HRP linked secondary antibodies (CST) were used at 1:5000 dilution. Proteins were visualized using ECL chemiluminescent substrate (Millipore). All results were normalized against β-tubulin, each measurement was performed in triplicate.

### Renal histology, immunohistochemistry, crystal detection and transmission electron microscopy

Kidneys were embedded in paraffin and sliced into 4-mm-thick sections, stained by hematoxylin and eosin (H&E), and Masson's trichrome for histological analysis. Immunohistochemical detection of inflammation was performed by using primary antibodies against macrophage marker F4/80 (1:200, #30325, CST), NLRP3 (1:200) and IL-1β (1:200). Renal fibrosis was detected by staining for collagen 1 (1:200, CST) and α-SMA (1:200, rabbit, 19,245, CST). Renal crystal sections were also obtained from absolute ethanol-fixed kidneys to detect uric acid crystals under polarized light. For transmission electron microscopy, kidneys were fixed with 2.5% glutaraldehyde, and post-fixed in 1% phosphate-buffered osmium tetroxide. After being embedded, sectioned, and double-stained with uranyl acetate and lead citrate, images were captured using transmission electron microscope (Tecnai G2 20 200 kV, FEI, USA).

### Statistics

Values are expressed as mean±standard deviation (±s.d.). All groups of animals were studied in parallel. Comparisons between different groups were performed using Student's *t*-test for unpaired samples. The level of significance was *P*<0.05. These analyses were conducted by using SPSS 21.0 and the Prism 6 software package (GraphPad, San Diego, CA, USA).
